# Health outcomes, education, healthcare delivery and quality – 3051. Profiles of diagnosis and treatment of asthma in the public health system in Brazil

**DOI:** 10.1186/1939-4551-6-S1-P220

**Published:** 2013-04-23

**Authors:** Janaina M Melo, Adriana S Moreno, Virginia PL Ferriani, Elcio Vianna, Marcos Borges, Persio Roxo, Ana Carla S Araujo, Luane M Mello, Rosa Ferreira, Jorgete M Silva, Patricia Stefanelli, Marcos R Gonçalves, Larissa P Oliveira, Andrea Cetlin, Luana B Queiroz, Rosangela Villela, Davi C Aragon, L Karla Arruda

**Affiliations:** 1 Medicine, School of Medicine of Ribeirao Preto - University of Sao Paulo, Ribeirao Preto, Brazil; 2 Pediatrics, School of Medicine of Ribeirao Preto - University of Sao Paulo, Ribeirao Preto, Brazil; 3 Social Medicine, School of Medicine of Ribeirao Preto, Ribeirao Preto, Brazil; 4 Pediatrics, University Center Barao de Maua, Ribeirao Preto, Brazil

## Background

Despite existence of several guideline resources, diagnosis and management of asthma remains a challenge in the real world. We aimed to evaluate diagnosis and treatment profiles among patients and physicians in the context of the Public Health System in Ribeirao Preto, a city in Southeast Brazil.

## Methods

ISAAC and ECRHS based questionnaires were used in a survey of 400 patients (109 children) who presented to the Emergency Department (ED) in the North District of the city with symptoms of asthma during the month of July 2011 (midwinter), to assess definition of asthma. A validated questionnaire was used to evaluate knowledge of asthma among physicians and other health care professionals working in Public Health Outpatient Clinics in the same area. Records of asthma medication delivered to the ED and of those provided to patients upon physicians´ prescription were analyzed within the period of August 2010 to July 2011.

## Results

Questionnaires for assessment of definition of asthma revealed that 82.5% and 89% of children and adults reported symptoms of asthma in the past twelve months, respectively. However, only 12.8% and 12.8%; and 21.3 and 19.6% of children and adults reported asthma ever or MD diagnosis of asthma, respectively. Among health care professionals, Physicians, Nurses and Pharmacists performed significantly better than Technical Professionals or Community Health Agents, with mean rates of correct responses to the questionnaire of 84.7%, 63.2% and 57.6%, respectively. Within one year, 835 20mL-vials of Fenoterol and 2,280 20mL-vials of Ipratropium Bromide were dispensed to the ED, as compared to 133 10mL-vials of Albuterol (F:A ratio 6.3) nebulization solutions. Physician´s prescription resulted in delivery of 2,876 units Beclomethasone spray; 3,205 units Albuterol spray, but also of 1,782 120ml-vials Albuterol syrup, 634 30-pack Albuterol oral tablets, and 1,570 30-pack oral Aminophylline, to outpatient users of the Public System.

## Conclusions

Asthma was under-diagnosed among patients seeking care for asthma symptoms in the ED. Treatment of asthma in the ED relied on use of Fenoterol and IpratropiumBromide, often in association,by nebulization. Outpatient treatment included prescription of oral medications for asthma. Diagnosis and treatment of asthma could be improved in this Public Health setting in Brazil.

